# President Garfield

**Published:** 1881-09

**Authors:** 


### EDITORIAL DEPARTMENT.

"If thou hast truth to utter, speak;
And leave the rest to God."

President Garfield.—The amount and character of the sympathy awakened by the dastardly attempt to assassinate Mr. Garfield was wonderful indeed, not only in our own country, north, south, east and west, but throughout the world; crowned heads even seemed to vie with one another in expressions of deep sympathy for him, and extended words of condolence to his immediate family in his hour of peril and of danger. In no part of the outer world, however, nor in our own country, indeed, did earlier or more earnest demonstrations and expressions of heartfelt grief, and sincere sympathy for him and his sorely afflicted household appear, than in the Southern States, "the sunny-land" of our own great country. The action there was prompt, spontaneous and general,and the clicking of the telegraph announcing the dastardly assault had hardly died away upon the tympanum, ere the people began dispatching words of sympathy for the suffering President, and utterances of deep condemnation of the would-be assassin's act, which jeopardised the life of the chief officer of this great Republic! The spontaneous outbursts of execration against the monster, Guiteau, and the heartfelt prayers of that people to the Almighty disposer of human affairs in the dark and trying hours of our country's peril, as the existence of its chief executive was oscillating between life and death, will furnish themes for song, and story, long ages after the intended victim and his would-be assassin have mouldered into dust.

The conduct of this people, during the entire crisis that succeeded the wounding of the President, marked a point of sublimity and moral grandeur that was never surpassed before in the history of the world! We see the entire people, from the Delaware to the Rio Grande, sublimely, grandly offering their libations of heartfelt sympathy for the wounded chief magistrate of our common country! Such conduct should cause the native American to feel proud that he was born in such a land, and the adopted citizen, also, to learn that there lies in the truly patriotic American heart a fountain which is *deeper* and *purer* than politics and the spoils of office!

But the pistol that prostrated the chief magistrate, and aroused the deepest sympathy as the report was echoed and re-echoed to the extremest limit and uttermost confines of the land, and its murderous missile placed his life in jeopardy, called forth, also, a fellow feeling in all true and patriotic hearts among the American people *everywhere;* and heartfelt earnest prayers from noble men and loving women ascended to the Throne of Grace for his preservation and early recovery; and from one extremity of this broad land to the other, political antagonisms and party strife yielded for a time, at least, to the benign touch and holy influence that makes all the world akin.

The very numerous and often conflicting opinions from physicians who had seen him, and others who were interviewed in regard to the President which reached us for many days, established one fact very conclusively to our mind, at least; that the tendency at the White House was to merge Mr. Garfield, the wounded man, into the wounded President of the United States. Had he been a man in the ordinary walks of life at that time, rather than the executive officer of a great country, we have no doubt that he would have gotten along quite as satisfactorily and comfortably under ordinary medical and surgical treatment. Gratitude to the All-wise disposer of events for his preservation restrains our pen from criticism now, however, but we may attempt to express our opinion upon the general management of the case in the future. H. L. B.

